# Magnetic Resonance Image Guided Focused Ultrasound Thalamotomy. A Single Center Experience With 160 Procedures

**DOI:** 10.3389/fneur.2022.743649

**Published:** 2022-02-18

**Authors:** Asad M. Lak, David J. Segar, Nathan McDannold, Phillip Jason White, Garth Rees Cosgrove

**Affiliations:** ^1^Department of Neurosurgery, Brigham and Women's Hospital and Harvard Medical School, Boston, MA, United States; ^2^Department of Radiology, Brigham and Women's Hospital and Harvard Medical School, Boston, MA, United States; ^3^Department of Chemistry and Physics, Simmons University, Boston, MA, United States

**Keywords:** focused ultrasound (FUS), thalamotomy, tremor, essential tremor (ET), Parkinson's disease (PD)

## Abstract

**Introduction:**

MRgFUS thalamotomy has gained popularity as an FDA approved, non-invasive treatment for patients with Essential Tremor and tremor predominant Parkinson's Disease. We present our initial clinical experience with 160 consecutive cases of MRgFUS thalamotomy and describe the clinical outcomes with long term follow-up.

**Methods:**

A retrospective chart review of all patients who underwent MRgFUS thalamotomy at our institution was performed. CRST Part A tremor scores were obtained pre-operatively and at each follow-up visit along with an assessment of side effects (SE). All patients had a post-operative MRI within 24 h to determine the location, size, and extent of the MRgFUS lesion.

**Results:**

One hundred and sixty unilateral MRgFUS Thalamotomies (Left, *n* = 128; Right, *n* = 32) were performed for medically refractory essential Tremor (*n* = 150) or tremor predominant Parkinson's disease (*n* = 10). Mean age at surgery was 75 Years (range: 48-93) and the mean skull density ratio (SDR) was 0.48 (range: 0.32-0.75; median: 0.46). In ET patients, both rest and postural tremor was abolished acutely and remained so at follow-up whereas intention tremor was reduced acutely by 93% below baseline, 87% at 3 months, 83.0% at 1-year, and 78% at 2 years. On post-operative day 1, the most common SE's included imbalance (57%), sensory disturbances (25%), and dysmetria (11%). All adverse events were rated as mild on the Clavien-Dindo Scale and improved over time. At 2-years follow-up, imbalance was seen in 18%, sensory disturbance in 10% and dysmetria in 8% patients. Mean clinical follow-up for all patients was 14 months (range: 1-48 months).

**Conclusion:**

MRgFUS thalamotomy is a safe and effective procedure for long term improvement of unilateral tremor symptoms, with the most common side-effects being imbalance and sensory disturbance.

## Introduction

Magnetic Resonance Image guided Focused Ultrasound (MRgFUS) thalamotomy has emerged as a novel treatment option for medically refractory tremor. The ability to ablate the intracranial target non-invasively, during an awake outpatient procedure, has made MRgFUS a reasonable treatment option for patients who are not suitable for or choose not to undergo an invasive surgical procedure. Since publication of the landmark randomized controlled trial (RCT) demonstrating the safety and efficacy of MRgFUS thalamotomy in unilateral Essential Tremor (ET) (1), reports from several centers have documented sustained benefit from the procedure at long term follow-up (2–5). Recent publications have attempted to identify key factors that may improve clinical outcomes following MRgFUS thalamotomy (4, 6–9). Skull Density Ratio (SDR), lesion location and lesion volume have all been reported as important factors that determine tremor outcomes and the adverse event profile (7, 10, 11). SDR has also been reported as a key factor in achieving therapeutic temperature at target site (8). Despite several publications reporting outcomes following MRgFUS, the current literature is limited by small sample size, heterogeneity in institutional protocols and studies involving multiple surgeons (2–4, 6).

In this retrospective observational study, we report our institution's experience with MRgFUS thalamotomy performed by the senior author (G.R.C) over a period of 4 years. We believe this represents a “real-world” clinical experience in a large number of patients undergoing this procedure and identifies areas for future advances in the field.

## Methods

After obtaining institutional review board approval, a retrospective chart review of all patients who underwent unilateral Magnetic Resonance Image guided Focused Ultrasound (MRgFUS) thalamotomy for medically refractory Essential tremor (ET) or tremor predominant Parkinson's disease from March 2016 to January 2021 was performed.

### Disease Characteristics

A detailed chart review was performed to extract demographics (age, gender), disease characteristics (family history, duration, tremor severity), treatment parameters [lesion location, sonication parameters (i.e., mean maximum temperature, mean maximum power, mean maximum energy, number of sonications), skull density ratio] and follow-up information (tremor scores in treated extremity, adverse events). Tremor scores in the treated extremity were documented using CRST Part A which rates tremor severity from 0 to 4, with 0 being no tremor and 4 being severe tremor (12). Tremor scores were documented at baseline, post-operative day 1, 3 months, 1 year, and later at annual follow-up. A systematic questionnaire for adverse events was documented at the same post-operative follow-up intervals.

### Surgical Procedure

Details of the surgical procedure have been published elsewhere (13). Briefly, on the day of the procedure, the patient's head was shaved, and a modified stereotactic frame was affixed low on the patient's skull after infiltration with local anesthetic. A flexible rubber gasket was placed over the frame posts and the patient's head firmly fixed to the MRI table. The space between the patient's head and the FUS transducer was then filled with circulating, degassed water and heavily T2-weighted images were obtained in sagittal, coronal, and axial planes. Standard stereotactic coordinates were used to locate the Ventral Intermediate Nucleus (Vim) of thalamus: 11 mm from the lateral wall of third ventricle, ¼ distance of the anterior commissure-posterior commissure (AC-PC) distance in front of PC and 1-2 mm above the intercommissural plane. Minor corrections to this initial target were made to adjust for individual patient anatomy. A baseline neurological examination was performed to assess speech, motor and sensory function, coordination and the magnitude of tremor with posture and intentional tasks (spiral, line drawing, and drinking from a bottle). Test sonications using lower temperatures were then performed to verify target alignment and determine the optimal lesion location. Once confirmed, higher temperature sonications were performed sequentially to ablate the Vim. Serial neurological examinations were performed after each sonication to assess for tremor improvement and side effects. No major change in methodology of FUS thalamotomy was undertaken except for altering the initial targeting to 1.5-2 mm above the intercommissural plane instead of at the intercommissural plane after the first few cases.

### Outcome Assessment

**Figure 1:**
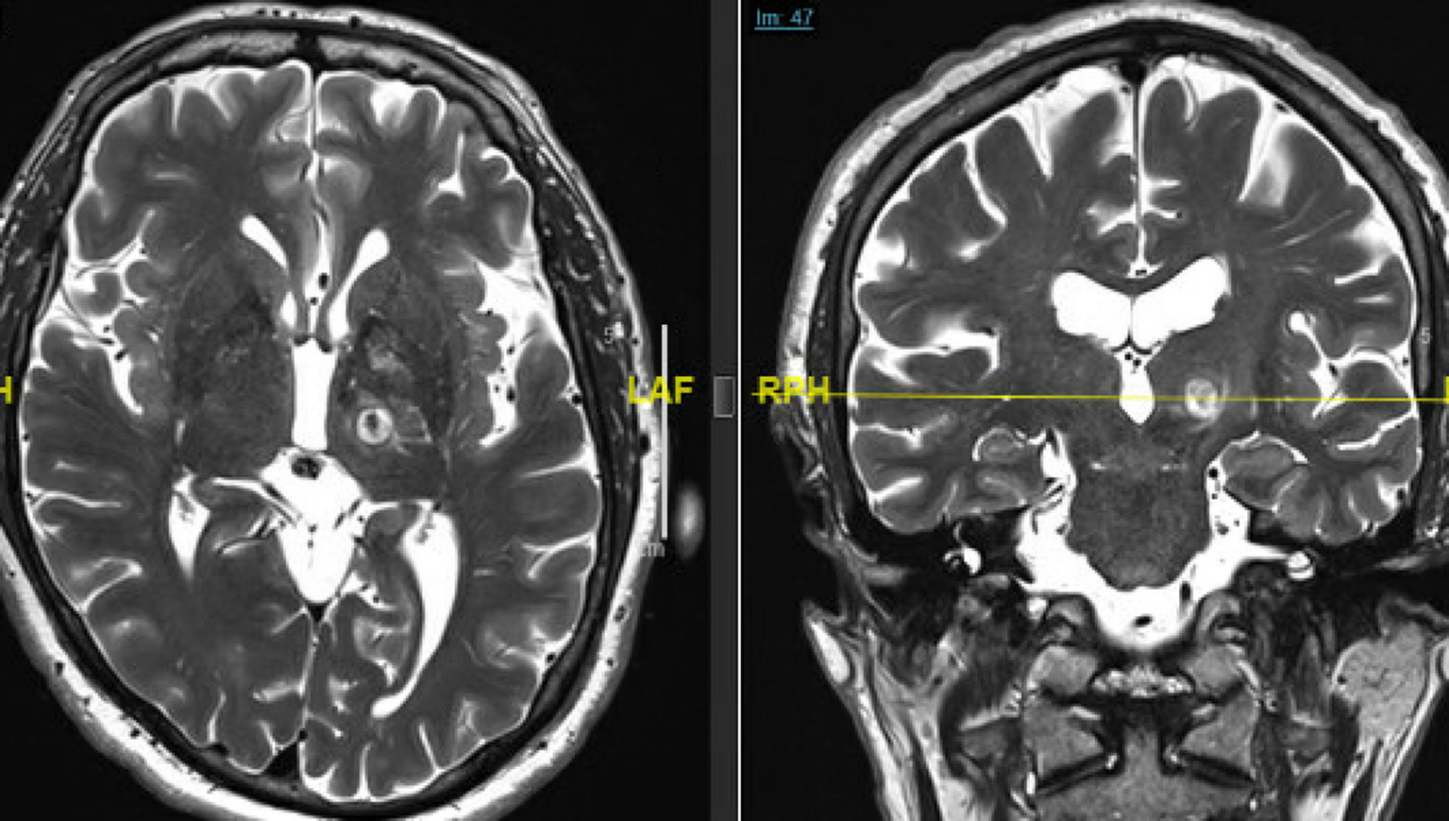
Immediate (24 h post-op) T2-weighted axial (left) and coronal (right) MRI scans demonstrating the size, shape, and location of a typical left Vim FUS thalamotomy. Note the edema extending around the lesion into the surrounding thalamus and the internal capsule.

Change in tremor scores were documented as percentage improvement from baseline and adverse events were recorded at each follow-up interval. An immediate MRI was obtained within 24 h post-operatively. Thin cut (2 mm) axial and coronal T2 slices were used for image analysis in this study (Figure 1). The center of the lesion was estimated on axial T2 images at the AC-PC plane and lesion volumes were then calculated by measuring the maximal distance from the center of the lesion along axial, coronal and sagittal axes. Wintermark zones 1 and 2 which represent coagulative necrosis and cytotoxic edema, respectively, and have been shown to correlate with permanent lesion were used for analysis of lesion volume (14). Wintermark zone 3 which represents vasogenic edema and is apparent on 24-h and 1-week MRI scans but resolves later was not used for calculation of lesion volume. In order to analyze the impact of SDR on tremor outcomes, the overall cohort was divided into two groups based on SDR. SDR <0.45 was called “low SDR” group and SDR ≥ 0.45 was called “high SDR” group. With only 10 patients treated for tremor predominant PD, only the subset of patients with a diagnosis of ET were included in the analysis of tremor outcomes. The entire cohort was however analyzed for the adverse events analysis.

### Statistical Analysis

Continuous variables are reported as mean ± standard deviation (range) and categorical variables as frequency (%). Comparison of tremor scores at each follow-up was performed using paired *t*-test. Comparison of percentage improvement in tremor scores in low SDR and high SDR groups was also performed using paired *t*-test.

## Results

### Demographics

A total of 160 cases were included in the study (Table 1). Treatment was aborted in one case due to a technical failure and inability to raise temperature, and one patient did not return after day 1 follow up, hence 158 patients were available for analysis of tremor outcomes. The mean patient age was 75 (range 55-93 years) and 68% were male. The overwhelming majority of patients had a diagnosis of ET (*n* = 150) whereas the rest had tremor predominant Parkinson's disease (*n* = 10). A positive family history was present in 68.75% patients and the mean duration of disease was 27.5 years. The mean SDR of entire cohort was 0.48 (median: 0.46; range 0.32-0.75).

**Table 1:** Demographics and clinical data of included patients (*n* = 160).

Age (years)	75.0 ± 7.50 (48-93)
Percentage males (%)	68.0% (*n* = 109)
Essential tremor	93.75% (*n* = 150)
Tremor-dominant Parkinson's	6.25% (*n* = 10)
Family history of tremor	68.75% (*n* = 110)
Mean duration from diagnosis (years)	27.5 ± 18.0 (2-70)
**Laterality of Thalamotomy**
Left	80.0% (*n* = 128)
Right	20.0% (*n* = 32)
**FTM intention tremor at follow up (ET)**	Significant difference from preop baseline (*p* < 0.0001)
Preop Baseline (*n* = 149)	3.39 ± 0.60 (2-4)
Day 1 (*n* = 148)	0.29 + 0.48 (0 – 2) (92.6%)
3 months (*n* = 110)	0.50 + 0.95 (0 – 4) (87.2%)
1 year follow-up (*n* = 101)	0.66 ± 1.08 (0 – 4) (83.1%)
2 year follow-up (*n* = 49)	0.87 ± 0.90 (0-3) (78.0%)
**Treatment parameters**	
Skull density ratio	0.48 ± 0.08 (0.32-0.75)
Lesion volume	335.45 ± 174.4 mm–^3^

### Tremor Scores

Left sided thalamotomy was most commonly performed (80%). The mean tremor score at baseline in ET patients was rest: 0.23 ± 0.55; posture: 2.72 ± 0.81; intention: 3.39 ± 0.60. The mean tremor score at baseline in tremor predominant PD was rest: 3.5 ± 0.52; posture: 2.8 ± 0.78; intention: 1.5 ± 1.18. Immediately following treatment, tremor scores in ET patients reduced sharply such that both rest and postural tremor scores declined to zero and remained so at long term follow-up. In addition, intention tremor was completely abolished in 107 patients on the first post-operative day. Intention tremor scores on post-operative day 1 (*n* = 148) were 0.29 ± 0.48, 0.50 ± 0.95 at 3 months (*n* = 110), 0.66 ± 1.08 at 1 year (*n* = 101), 0.87 ± 0.90 at 2 years (*n* = 49), 1.25 ± 0.57 at 3 years (*n* = 8) and 1 ± 0.63 at 4 years (*n* = 6) follow-up. At 1-year follow-up, nine patients had lost > 50% of their treatment benefit and at 2 years follow-up, five additional patients developed recurrence of tremor. In patients with tremor predominant PD, tremor scores also declined to zero and remained so till 3-months follow-up. At 1 year follow up (*n* = 4), mean rest tremor scores in PD patients were 0.75 and at 2 years (*n* = 2) it was 1.00.

### High SDR vs. Low SDR Group

**Figure 2:**
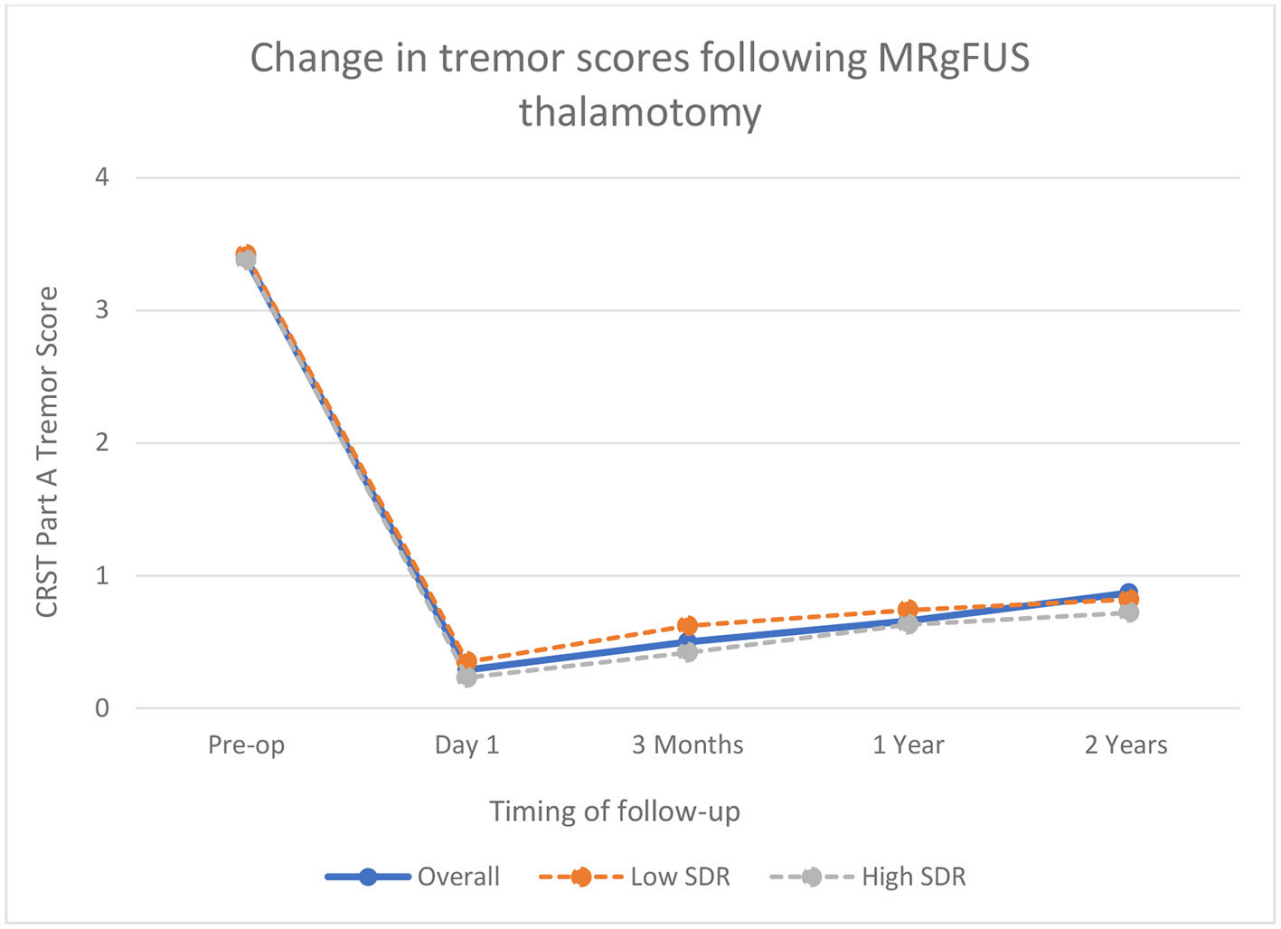
Change in tremor scores following MRgFUS thalamotomy in Essential Tremor patients.

In comparing ET patients based on SDR, the percentage improvement in tremor scores was slightly higher in high SDR group at each follow-up interval [93% on day 1, 87% at 3 months, 81.5% at 1 year and 79% at 2 years as compared to 90% on day 1, 82% at 3 months, 78.5% at 1 year and 76% at 2-years] but this finding was statistically non-significant (Figure 2). The high SDR group had on average larger lesions than the low SDR group [310.5 vs. 262.0 mm^3^], lower mean maximum energy [17242.0 vs. 26253.5 J], lower mean maximum power [936.0 vs. 1048.5 Watts] and higher mean maximum temperature [61.0vs. 57.5°C]. At 1-year follow-up, there were five patients in each group who had lost treatment benefit and were back to baseline tremor scores.

### Adverse Events

**Figure 3:**
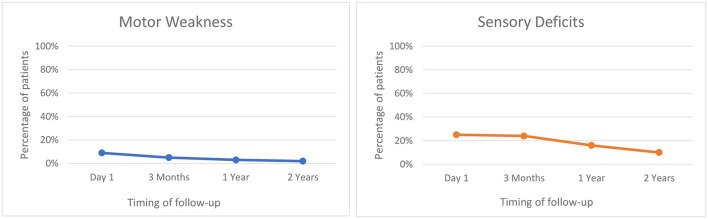
Evolution of adverse events following MRgFUS thalamotomy.

Adverse events following treatment were common (Table 2). On post-operative day 1 (*n* = 160), the most common adverse events were gait imbalance (56.8%) followed by sensory deficits (25%), dysarthria (18.75%), dysmetria (11.25%), motor weakness (8.75%), headache (3.12%), dysgeusia (0.62%), and others (2.5%). The majority of adverse events were transient (Figure 3) such that at 3-months follow up (*n* = 116), gait imbalance was seen in 25.8% patients, sensory deficits in 24.15%, dysarthria in 7.75%, dysmetria in 10.34%, motor weakness in 5.17%, and dysgeusia in 7.75% cases. At 1 year follow up (*n* = 105), most common adverse events were sensory deficits (16.2%) followed by gait imbalance (14.28%), dysmetria (6.66%), dysarthria (5.71%), motor weakness (2.85%), and dysgeusia (2.85%). The similar trend continued at 2 years follow-up (*n* = 51) where the most common adverse events were gait imbalance (17.5%), sensory deficits (10.0%), dysmetria (8.0%), dysgeusia (4.0%), and motor weakness (2.0%).

**Table 2:** Adverse events.

**Adverse events**	**1-day post-op (*n* = 160)**	**3-months post-op (*n* = 116)**	**1-year post-op (*n* = 105)**	**2-years post-op (*n* = 51)**
Motor weakness	14 (8.75%)	6 (5.17%)	3 (2.85%)	1 (2.0%)
Face	5 (3.12%)	-	-	-
Limb	7 (4.37%)	6 (5.17%)	3 (2.85%)	1 (2.0%)
Face and limb	2 (1.25%)	-	-	-
Dysarthria	30 (18.75%)	9 (7.75%)	6 (5.71%)	1 (2.0%)
Sensory deficits (Paresthesia/Numbness)	40 (25.0%)	28 (24.15%)	17 (16.2%)	5 (10.0%)
Orofacial	27 (16.87%)	20 (17.25%)	11 (10.5%)	1 (2.0%)
Orofacial and finger	10 (6.25%)	5 (4.30%)	6 (5.71%)	3 (6.0%)
Fingers	3 (1.87%)	3 (2.5%)	-	1 (2.0%)
Gait imbalance	91 (56.87%)	30 (25.8%)	15 (14.28%)	9 (17.5%)
Dysgeusia	1 (0.62%)	9 (7.75%)	3 (2.85%)	2 (4.0%)
Dysmetria	18 (11.25%)	12 (10.34%)	7 (6.66%)	4 (8.0%)
Headache	5 (3.12%)	-	-	
Others (hypotension/ lightheadedness, somnolence, new onset LE tremor)	4 (2.5%)	-	-	

## Discussion

In this retrospective study of 160 patients undergoing unilateral MRgFUS thalamotomy for severe, intractable essential tremor, we have demonstrated marked improvement in tremor scores of the treated arm in >90% of patients. This improvement was sustained at long-term follow-up as highlighted by sustained ~80% improvement in tremor scores at 2-years follow-up. Although adverse events were common, the majority were transient and had resolved or improved substantially at long-term follow-up. We also demonstrated that patients with SDR <0.45, i.e., “low SDR” group had comparable improvements in tremor scores as the patients with SDR ≥ 0.45. Another reassuring finding in our study is the sustained improvement in tremor scores at long-term follow-up. Notably, our long-term tremor outcomes are slightly better than what is currently reported in the literature (3, 4, 15).

SDR has been found to correlate with the ability to achieve therapeutic temperatures at target site and current recommendations suggest an SDR ≥ 0.45 for successful ablation (16). Many centers feel this recommendation is somewhat arbitrary and offer MRgFUS to patients with an SDR <0.45. A recently published trial in a Japanese population demonstrated positive outcomes in a population with lower average SDR than other studies (2). This study highlighted that patients with lower SDR had lower maximum temperature than the high SDR group, but the temperature achieved was still sufficient to provide clinical benefit. Two additional studies have also concluded that SDR does not affect clinical outcome despite being correlated with a lower maximum temperature at target site (16, 17). Our results align with the previously reported studies and highlight that percentage improvement in tremor scores at long-term follow-up was comparable between the two groups.

Due to the proximity of MRgFUS lesion to key adjacent thalamic nuclei and the internal capsule, adverse events following MRgFUS thalamotomy are frequent with the incidence of adverse events ranging from 10 to 60% (18). In our experience, the most commonly encountered adverse events were gait imbalance and sensory deficits which is consistent with earlier studies. Notably, all the adverse events following MRgFUS thalamotomy in our study were mild and classified as Clavien-Dindo Grade I (19). Previous studies like ours have identified several key factors related to the occurrence of complications. Lesion location, volume and extent have all been identified as important factors determining the occurrence of complications (7, 11). In a previously published study, we demonstrated that patients with any adverse event had significantly larger lesion (300 vs. 229mm^3^) and had more inferior and lateral lesion margins (11). Extension of the lesion laterally into the internal capsule can lead to contralateral weakness or dysarthria. Posterior extension can lead to sensory deficits in the face or fingers. Inferior extension can lead to imbalance and dysmetria (11, 13). Lesion location and volume are important considerations for both enduring therapeutic benefit and the occurrence of complications. Our lesions were consistently located on our specified target but were significantly larger than previously reported at other centers. Some studies have suggested a particular lesion volume threshold to achieve maximum tremor benefit while avoiding complications (7). A thalamic lesion volume of at least 40 mm^3^ is necessary to achieve tremor benefit (20). However, larger lesion volumes have been suggested to achieve better tremor control (21). Notably, larger lesions volumes have also been found to be associated with higher risk of adverse events (7, 11). Other studies have suggested using a tractography based approach to localize the optimal target which can optimize clinical outcomes although these techniques were not used in any of our cases (22). Despite our larger average treatment volume, our complication rates were consistent with all previously reported studies. Moreover, every patient was questioned for adverse events in a standardized fashion at each follow-up interview to more accurately reflect the incidence of complications in our series. Nevertheless, future efforts should explore detailed post-operative MRI lesional analysis to help determine the optimal lesion location and volume to maximize long term therapeutic outcomes and minimize complications. Advances in MRI tractography and imaging may optimize targeting of Vim in the future as well.

### Limitations

The major limitations of this study are due to its' retrospective nature and the fact that post-operative evaluations were performed by an unblinded observer which could have led to a risk of positive reporting bias. Since the goal of the study was to present a “real-world” clinical experience hence standardized tremor scales were not utilized during routine follow-up evaluations. Moreover, the small number of PD patients in our series limited our ability to perform meaningful tremor outcomes analysis in this subset of patients.

## Conclusion

MRgFUS thalamotomy is a safe and effective procedure for unilateral tremor symptoms. Adverse events following the procedure are common but generally mild and transient in the majority of cases. Future work should explore the optimal MRgFUS lesion location, volume and extent in order to maximize long term tremor control and minimize complications.

## Data Availability Statement

The raw data supporting the conclusions of this article will be made available by the authors, without undue reservation.

## Ethics Statement

The studies involving human participants were reviewed and approved by Brigham and Women's Hospital IRB. Written informed consent for participation was not required for this study in accordance with the national legislation and the institutional requirements.

## Author Contributions

AL and DS performed the retrospective analysis of clinical material and initial formulation of manuscript under the direction of GC. GC performed all procedures assisted by magnetic resonance physicists, NM and PW. All authors contributed to the writing and editing of the manuscript.

## Funding

GC has received clinical research support from Insightec.

## Conflict of Interest

The authors declare that the research was conducted in the absence of any commercial or financial relationships that could be construed as a potential conflict of interest.

## Publisher's Note

All claims expressed in this article are solely those of the authors and do not necessarily represent those of their affiliated organizations, or those of the publisher, the editors and the reviewers. Any product that may be evaluated in this article, or claim that may be made by its manufacturer, is not guaranteed or endorsed by the publisher.
